# Role of Connexins 30, 36, and 43 in Brain Tumors, Neurodegenerative Diseases, and Neuroprotection

**DOI:** 10.3390/cells9040846

**Published:** 2020-03-31

**Authors:** Oscar F. Sánchez, Andrea V. Rodríguez, José M. Velasco-España, Laura C. Murillo, Jhon-Jairo Sutachan, Sonia-Luz Albarracin

**Affiliations:** Department of Nutrition and Biochemistry, Pontificia Universidad Javeriana, 110911 Bogota, Colombia; a_rodrigueza@javeriana.edu.co (A.V.R.); jose.velasco@javeriana.edu.co (J.M.V.-E.); laura.murillo@javeriana.edu.co (L.C.M.); jsutachan@javeriana.edu.co (J.-J.S.)

**Keywords:** connexins, gap junctions, microglia, astrocytes, neurons, neuroprotection, neurodegenerative diseases, epigenetics

## Abstract

Gap junction (GJ) channels and their connexins (Cxs) are complex proteins that have essential functions in cell communication processes in the central nervous system (CNS). Neurons, astrocytes, oligodendrocytes, and microglial cells express an extraordinary repertory of Cxs that are important for cell to cell communication and diffusion of metabolites, ions, neurotransmitters, and gliotransmitters. GJs and Cxs not only contribute to the normal function of the CNS but also the pathological progress of several diseases, such as cancer and neurodegenerative diseases. Besides, they have important roles in mediating neuroprotection by internal or external molecules. However, regulation of Cx expression by epigenetic mechanisms has not been fully elucidated. In this review, we provide an overview of the known mechanisms that regulate the expression of the most abundant Cxs in the central nervous system, Cx30, Cx36, and Cx43, and their role in brain cancer, CNS disorders, and neuroprotection. Initially, we focus on describing the Cx gene structure and how this is regulated by epigenetic mechanisms. Then, the posttranslational modifications that mediate the activity and stability of Cxs are reviewed. Finally, the role of GJs and Cxs in glioblastoma, Alzheimer’s, Parkinson’s, and Huntington’s diseases, and neuroprotection are analyzed with the aim of shedding light in the possibility of using Cx regulators as potential therapeutic molecules.

## 1. Introduction

Cellular communication, through dynamic and often bi-directional interactions between glial and neuronal cells, is fundamental for the development and normal function of the central nervous system (CNS) [1]. In this regard, intercellular communication is vital not only to detect changes from the extracellular matrix, but also from soluble mediators present in the microenvironment and other neighboring cells to maintain homeostasis. In the CNS, intercellular communication can occur through synaptic transmission, paracrine signaling, and electrical coupling [1]. Gap junctions (GJs) are complex structures constituted by homomeric or heteromeric hexamer of connexin (Cx) hemichannels (HCs) located in the apposed plasma membranes of two adjacent cells [2]. Cxs are characterized by having nine domains, four alpha-helical transmembrane domains, two extracellular loops, and a unique intracellular loop, cytoplasmatic carbonyl and amine terminals [3]. 

In the CNS, GJs allow the intercellular transport of small molecules by passive diffusion. These molecules are typically Ca^2+^, Na^+^, and K^+^ ions, metabolic molecules, such as glucose, adenosine triphosphate (ATP), and cyclic adenosine monophosphate (cAMP), neurotransmitters like glutamate, and gliotransmitters [4,5]. In mammals, the different cell types present in the brain express over ten different Cx proteins, which make it an organ with diverse and complex intercellular communication. For instance, in trigeminal ganglia neurons, mRNA of Cx26, Cx36, Cx40, and Cx43 were detected [6]. However, in neurons, protein expression was mainly limited to Cx26, Cx36, and Cx40 with barely detected levels of Cx43 [6]. In addition, during the neuron life cycle, the presence and protein expression level of these Cxs varies [7]. For instance, in mouse, a dramatic reduction in Cx36 protein levels is observed from the embryonic neurogenesis to the post-natal maturation [7]. Similarly, mRNA and protein of Cx26, Cx30, and Cx43 have been detected in astrocytes; however, their expression is heterogeneous throughout the CNS [8,9]. For example, protein levels of all three Cxs are abundant in subcortical regions, while in the cerebral cortex, Cx30 and Cx26 protein levels are moderate and low, respectively [10]. Furthermore, Cx30 is not detectable in astrocytes from the white matter [11]. These results suggest functional differences in GJ based on different functional requirements among CNS regions. On the other hand, different mRNA and protein levels of Cx32, Cx36, and Cx43 have been found in microglia depending on its state, resting or activated, and the effector that triggers this state shift such as tissue damage or a pathological state [8,9]. For instance, microglia in the resting state highly expresses Cx32 and Cx36 mRNA and protein [12,13], and Cx43 mRNA or protein is rarely detected [14,15,16]. Conversely, in activated microglia, it has been observed the overexpression of Cx32, Cx36, or Cx43 proteins [17,18,19,20].

**Figure 1:**
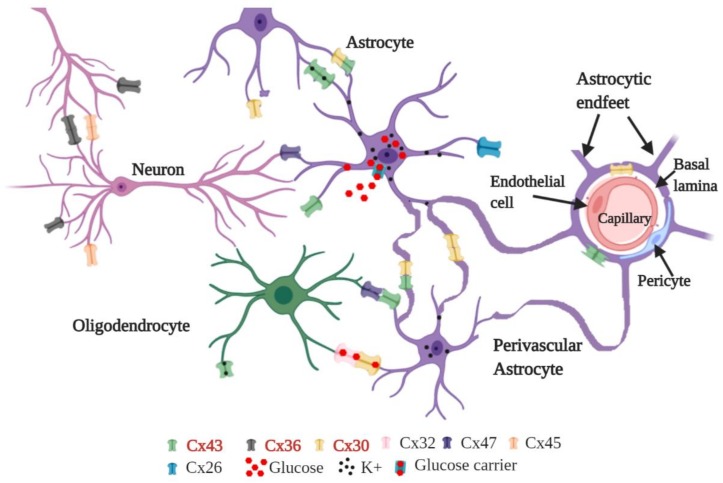
Schematic representation of the distribution of Cxs in the CNS. Cx30 and Cx43 are mainly present in astrocytes; while Cx36 is mainly expressed in neurons.

Despite the relevance that all Cxs have for intercellular communication in the brain, recent studies have unveiled important roles for Cx30, Cx36, and Cx43 in cell homeostasis, neurodegeneration, and neuroprotection. Cx30, which is one of the main Cxs present in astrocytes, has a fundamental role in astrocyte-astrocyte communication, nutrients transport, and K^+^ buffering [21]. In animal models, specifically in mice, Cx30 is involved in cognition and behavior [22]. For instance, knocking out Cx30 modifies the response of mice to novel environments and impairs the recognition of novel objects [23]. Additionally, it has been shown that Cx30 modulates glutamate transport in the hippocampus, highlighting the importance of this Cx in regulating the excitatory synaptic transmission [24]. On the other hand, Cx36 is thought to play an essential role in neuronal development because its expression reaches a maximum when extensive inter-neuronal coupling takes place [25]. Indeed, Cx36 is the primary Cx in neurons, and its knockout (KO) leads to almost a complete loss of neuronal GJ coupling in the mature CNS, which primary role is the modulation of synchronized oscillatory activity in the CNS [26,27,28]. Furthermore, Cx36 KO revealed that electrical coupling has an important role in learning and memory [29], in high-frequency and γ-oscillation in the hippocampus [28,30], and synchronized activity in the inferior olivary nucleus, inferior olive, and cerebellum [31,32,33]. In contrast to Cx30 and Cx36, which are restricted to astrocytes and neurons, respectively, Cx43 protein is mainly present in astrocytes and active microglia [34,35], making this the most important Cx for the GJ coupling between astrocytes and HCs present in the microglia. Furthermore, Cx43 is also present in the endothelial wall of blood vessels; however, Cx43 protein levels are low in healthy blood vessels [36]. Figure 1 shows a schematic representation of the distribution of Cx30, Cx36, and Cx43 in different cells of the CNS.

On the other hand, compiling evidence suggests that deregulation of these Cxs correlates with neurodegenerative diseases. For example, in rat and mouse models of Parkinson’s disease (PD), astrocytes in the striatum showed an increased level of Cx30 [37,38]. Moreover, while neuronal survival is reduced in damaged neuronal networks due to excessive coupling, in Cx36 KO animals, neuronal death is reduced [39]. Conversely, in in vitro assays, the acute application of amyloid-β_25–35_ (Aβ_25–35_) increases the activity of Cx36 along with pannexin 1 channels leading to potential neuronal death [40]. Additionally, changes in protein and mRNA levels of Cx43 have been correlated with Alzheimer´s disease (AD) in human post-mortem brain tissue, where reactive astrocytes are overexpressing this Cx co-localize with ~80% of amyloid plaques.

Given the relevance of Cx30, Cx36, and Cx43 in the CNS, we provide an overview of the regulatory mechanisms involved in the expression of these Cxs and their role in brain cancer, CNS disorders, and neuroprotection. In particular, we focus on recent advances in understanding the mechanisms that explain the role of selected Cxs in CNS disorders and neuroprotection, which can shed light on the development of novel therapeutic opportunities.

## 2. Connexins: Gene Structure and Transcriptional Regulation via Epigenetic Mechanisms

Among the different Cxs, there are differences in their gene structure that might underlie cell-type-specific expression patterns. Understanding then the molecular basis of the transcription control of Cxs is a crucial point to figure out the clinical effects of their dysregulation. Although several studies have unveiled the control that different transcription factors exert on Cxs expression (review in [41,42]), gene transcription control via epigenetic machinery has gained attention in clinical contexts. For instance, in cancer and neurodegenerative diseases, altered gene expression patterns of essential genes, such as oncogenes and tumor suppressor genes, have been correlated with the aberrant regulation of the epigenetic machinery and thus of the different epigenetic traits [43,44]. Then, understanding how aberrant changes in the epigenetic machinery affect Cxs transcription and their role in the onset of relevant diseases is of interest. We provide here an overview of the relevant traits associated with the gene structure and the epigenetic control of transcription of selected Cxs, namely Cx30, Cx36, and Cx43, in brain cells.

### 2.1. Gene Structure

The general gene structure of Cxs is considered to be constituted by a 5´untranslated region (5´-UTR) on exon 1 separated from the Cx coding region and subsequent 3´-UTR [42,45]. However, accumulating evidence has shown that this simple gene structure presents several variations. For instance, several Cxs present alternative splicing forms, implying that different 5´-UTRs can be spliced in a consecutive or alternate manner. It has also been detected the presence of introns in the coding region of several Cxs [42,45].

**Figure 2:**
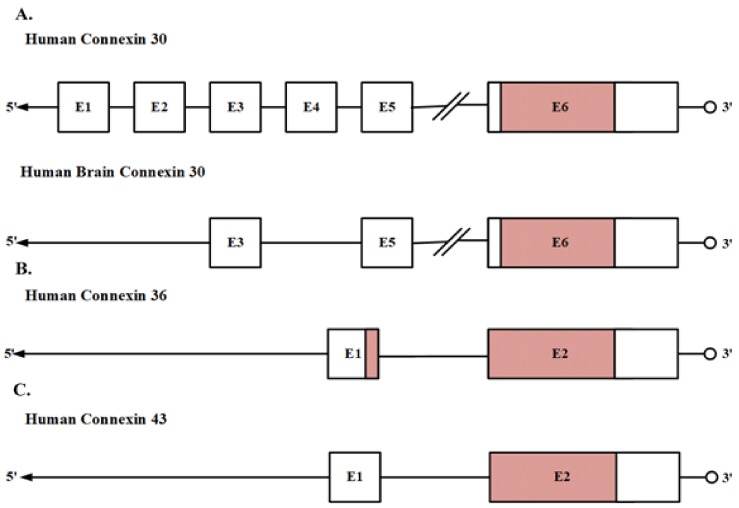
Schematic representation of the genomic structure of (**A**) GJB6 (Cx30) on chromosome 13; in the human brain it has been found that GJB6 has only the non-coding exons 3 and 5 and the coding exon 6. (**B**) GJD2 (Cx36) on chromosome 15, and (**C**) GJA1 (Cx43) on chromosome 6. Each box represents an exon, and solid red boxes represent the coding regions.

Under this perspective, Cx30, Cx36, and Cx43 have variations in their gene organization compared to this simple model. We focus here in the observed gene organization of these Cxs obtained from human brain cDNA. Cx30 is encoded by the Gap Junction Protein Beta 6 (GJB6) gene present in chromosome 13q12, which has been reported to have six different exons. Exons 1 to 5 are non-coding exons located upstream of the coding exon 6. However, the cDNA from the human brain only displays two non-coding exons, 3 and 5, and the coding exon 6. Some of the Cx30 exons can be alternatively spliced, leading to high variability in the 5´non-coding region of the Cx30 transcripts [46]. Besides, tissue-specific splicing has been suggested since the non-coding exon 3 is present only in nervous system cells [46]. Cx36 is encoded by the Gap Junction Protein Delta 2 (GJD2) gene present in chromosome 15q14, which is interrupted by a 1.05-kb intron, which is located 71 bp after the translation initiation site. This intron separates the amino-terminal region domain from the first transmembrane region [47]. Cx43 is encoded by the Gap Junction Protein Alpha 1 (GJA1) gene present in chromosome 6q22-q23, which is composed of two exons with an intervening intron of 11 kb. The first exon contains most of the 5´-UTR while the second exon contains 16 bp of the 5´-UTR, followed by 1149 bp of the coding sequence, and about 1732 bp of 3´-UTR. In addition, there is a GJA1 pseudogene located within chromosome 5 at 5q21-5q22 [48] (Figure 2). 

### 2.2. Transcription Regulation via Epigenetic Mechanisms

**Figure 3:**
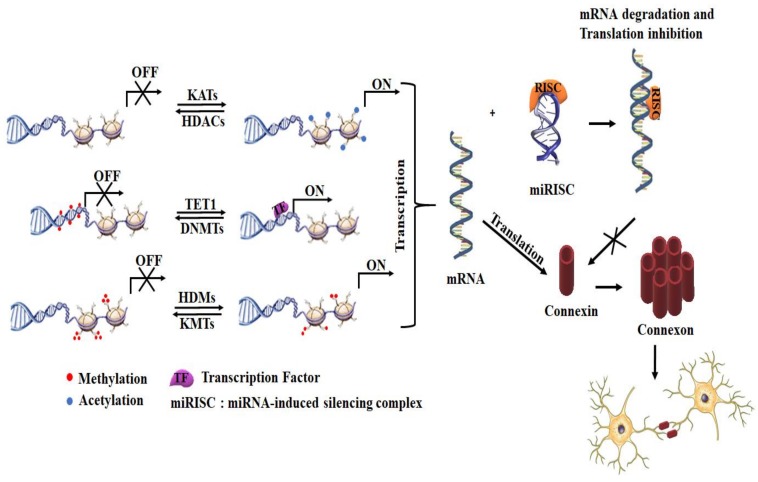
Regulatory epigenetic processes on Cx expression. Histone acetylation, DNA and histone methylation, and microRNAs (miRNA) are presented as the main studied epigenetic controls over Cxs gene regulation. Gene expression is correlated with histone acetylation, low DNA methylation in the promoter region, while gene repression correlates with high DNA methylation in the promoter region and low histone acetylation levels. Histone methylation control on gene expression is residue-specific and also depends on the grade of methylation of the residue. Lysine can be mono-, di- or tri-methylated. KATs: lysine acetyltransferases; HDACs: histone deacetylases; TET1: Tet-Eleven Translocation 1 enzyme, main DNA demethylases in mammals; DNMTs: DNA methyltransferases; HDMs: histone demethylases; KMTs: lysine methyltransferases.

Epigenetic machinery, together with specific transcription factors, controls Cxs expression, making it possible to find Cxs distinctively expressed in different mammal cells [49]. However, there are many cell types that lack GJ, such as mature sperm, erythrocytes, differentiated skeletal muscle cells, among others [49]. Cxs are also found in various cell types like Cx43, which is one of the major GJ in astrocytes; meanwhile, Cx36 found mainly in neurons. The major epigenetic mechanisms involved in the regulation of Cx expression are DNA methylation (^me^CpGs), histone post-translational modifications (PTMs), and posttranscriptional gene silencing by microRNAs (miRNAs). Collectively, they create epigenetic landmarks that dictate the compactness of chromatin and transcriptional state of cells. Based on that, chromatin can be divided into an active (eu-chromatin/transcription “on”) and inactive (hetero-chromatin/transcription “off”) chromatin [50]. The presence of selected epigenetic modifications at least partially drives the formation of eu- and hetero-chromatin. DNA hypermethylation of gene promoters has been typically linked to transcription silencing. This DNA methylation is dynamically control by DNA methyltransferases (DNMTs) and DNA demethylases enzymes (like the ten-eleven translocation methylcytosine dioxygenase 1 (TET1)) [51]. Among the different histone PTMs, acetylation of histones can lead to decompaction of chromatin [52] and is abundant at transcriptional starting sites of actively transcribed genes [53,54,55]. Histone acetylation is regulated by the activity of lysine acetyltransferases (KATs) and histone deacetylases (HDACs), which acetylates and deacetylates lysine residues in histones, respectively [56,57]. Histone methylation is another important PTMs. The main amino acid residue methylated in histones is lysine, which can be mono-, di- or tri-methylated. Contrary to lysine acetylation, the lysine methylation level confers different regulation properties in a residue-specific manner. For instance, the presence of tri-methylated lysine 4 of histone H3 (H3K4me3) in the gene promoter region is associated with transcriptional activation, while active enhancers are enriched with mono-methylated H3K4 [58]. This histone methylation is modulated by histone lysine methyltransferases (KMTs) and histone demethylases (HDMs) [59]. The other epigenetic control can be exerted via miRNAs that are short single-stranded non-coding RNAs of ~22-nucleotides. These miRNAs regulate the gene expression at a post-transcriptional level of a specific mRNA by a Watson–Crick base pairing between the miRNA “seed region” and mRNA sequences. Once the miRNA is bound to an argonaute protein family member (AGO), it serves as a guide for the core silencing complex, known as miRNA-induced silencing complex (miRISC), to bind complementary sequences typically found in the 3’-UTR of mRNAs, reducing protein expression levels via mRNA degradation, translational inhibition, or transient mRNA sequestration [60,61] (Figure 3).

Dysregulation of epigenetic machinery has been typically associated with the development of cancer and neurodegenerative diseases. There is compiling evidence about the DNA methylation-driven suppression of Cx expression in various human carcinomas, as well as the overexpression of Cxs in cancer cells treated with DNMT inhibitors (DNMTi) that results in the enhancement of GJ activity, though this effect is connexin-specific and cell type-dependent (reviewed in [42,62]). To the best of our knowledge, only one study using neuronal cells from mouse has evaluated the effect of DNMTi on the expression of the Cxs of interest. In this report, the neuronal cell line SN56 and pituitary cell line AtT20 from mouse were used to study the effect of DNMTi 5-azacytidine on the expression of Cx36, which is a target gene of REST (RE-1 silencing transcription factor) [63]. The results showed no effect on the expression of Cx36, suggesting that the regulation of the Cx36 gene can occur via other epigenetic marks like histone PTMs, as discussed later. A recent work, in the neuropsychiatric field, attempted to correlate changes in DNA methylation levels of the Cx43 gene intron region with major depression disorders (MDD) [64]. This study, based on previous findings where levels of Cx43 and Cx30 in the prefrontal cortex of subjects with depression that committed suicide were low [65], did not find differences in DNA methylation levels between controls and MDD subjects, suggesting no association between Cx43 gene methylation and the studied depression phenotype. In contrast, hypermethylated CpGs in the promoter region of the Cx30 gene were found in high-grade glioblastomas (grade III and IV) rather than in low-grade glioblastomas (grade I and II) [66]. Interestingly, this hyper-methylated region corresponds to the recognition sites for Sp1 and Ap2 transcription factors [66] and correlates with a progressive downregulation of Cx30 mRNA with the glioblastoma grade. Similarly, downregulation in Cx43 mRNA was observed irrespectively of the methylation level found in the intron region of the Cx43 gene for the different glioblastoma grades [66].

Evaluation of the effect of histone PTMs and their related epigenetic machinery on Cx expression has not been widely explored, the effect of HDAC inhibitors (HDACi), such as trichostatin A (TSA), sodium butyrate, and 4-phenylbutyrate, on Cx gene expression being the most studied. HDACi have been evaluated primarily in cancer cells, leading to a global histone hyper-acetylation, enhancing Cx expression and gap junctional intercellular communication (GJIC) [62]. Interestingly, it has been found that cell-specific determinants are essential for the HDACi to alter the Cx gene transcription, along with the presence of active or repressive epigenetic marks. For instance, TSA enhanced Cx36 expression in pancreatic cells, which express REST, a transcription repressor of neuronal genes present in non-neuronal cells [63]. However, TSA did not trigger the expression of Cx36 in either of the mouse cell lines SN56 and AtT20. This cell type-specific regulation of target genes was further explained by the presence of the repressive mark H3K9me2 in the neuron-restrictive silencer element (NRSE) site of Cx36 in AtT20 cells [63].

On the other hand, how histone methylation affects the expression of Cxs in brain tissues has not been extensively studied. To the best of our knowledge, only one study has reported how altered chromatin states associated with H3K9me3 enrichment causes the downregulation of Cx30 in astrocytes from cortical and subcortical brain regions of depressive patients [67]. Like DNA methylation and histone PTMs, miRNAs have shown to regulate Cx expression. Despite that more than 200 miRNAs [68,69] in mammalian neurons and astrocytes have been identified, there are only a few reports relating miRNA to Cx expression in neurons and astrocytes, and these are mainly in brain cancer. In the peripheral nervous system, when chronic constriction injury of the sciatic nerve causes neuropathic pain, the overexpression of Cx43 and downregulation of miRNA-1 has been found, suggesting that in neuropathic pain, there is a regulation in the expression of Cx43 by miRNA-1 [70]. In brain tumors, specifically in gliomagenesis, it was found that miRNA-221/222 acts as an oncogenic miRNA by targeting Cx43, reducing its expression, and promoting glioma growth [71]. Furthermore, a low Cx43 expression has been correlated with a poor prognosis in glioma patients [72]. Interestingly, no studies associating miRNAs and expression of Cx30 and Cx36 have been reported. Nevertheless, other reports have evaluated changes in the expression of Cxs induced by miRNAs in other tissues, mainly muscle and heart, reviewed elsewhere [42,62].

## 3. Life-Cycle Modulation of Connexins by Post-Translational Modifications

Post-translational modifications (PTMs) generally occur to control the activity of different proteins [73]. In the case of Cxs, PTMs are involved in the modulation of their life-cycle, including synthesis and degradation, trafficking, protein–protein interactions, and GJ channel gating [41]. Cxs have intracellular domains with relatively unstructured nature, making this a perfect scenario for conformational changes induced by PTMs. Most Cxs have several consensus sites that can be phosphorylated, acetylated, S-nitrosylated, ubiquitinated, and SUMOylated, among others. Despite that the latest PTMs were discovered almost three decades ago, such as the ubiquitin-mediated degradation system dated from the early 1980s, and related to SUMO, dated from the late 1990s [74,75], studies reporting the role of different PTMs on different Cxs remain scarce. We highlight here some of the most critical aspects related to PTMs of Cx30, Cx36, and Cx43, focusing on the PTMs present on Cx43, which is one of the most studied Cx. The reader is then referred to recent and comprehensive reviews on Cx PTMs [76,77,78,79,80].

**Figure 4:**
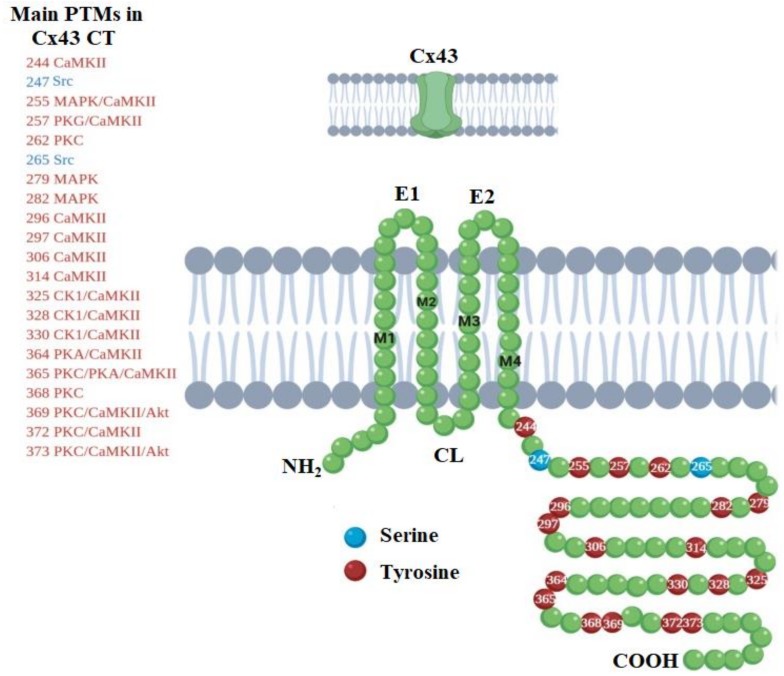
Schematic representation of Cx43 embedded in a cell membrane. Main PTMs present in Cx43 CT are listed. E1 and E2 are external loops, CL is the cytoplasmic loop, and M1 to M4 are the transmembrane domains.

The C-terminus (CT) of Cx43 is fundamental for the correct function of Cx43 GJs, being subject to extensive PTMs such as phosphorylation, acetylation, s-nitrosylation, ubiquitination, and SUMOylation [81]. Among the different PTMs, phosphorylation is the best known. Cx43 CT is mainly phosphorylated on serine residues, although tyrosine and threonine can also be phosphorylated. For instance, it has been reported that phosphorylation of Ser279 and Ser282 by the mitogen-activated protein kinase (MAPK) promotes the endocytosis of Cx43 GJs [82]. Conversely, the phosphorylation of Ser373 has been proposed to be an upstream gatekeeper of a cascade of PTMs involved in GJ endocytosis. Due to that, this phosphorylation prevents protein kinase C (PKC) or MAPK to phosphorylate Ser368 or Ser255, which are involved in the loss of GJIC and Cx43 ubiquitination and endocytosis [83]. On the other hand, GJ assembly has been associated with the phosphorylation induced by casein kinase 1 (CK1) on Ser325, Ser328, and Ser330 [84]. During mitosis, Ser255 and Ser262 are phosphorylated by the cyclin-dependent kinase 1 (CDK1), which correlates with the downregulation of GJIC and increased endocytosis of Cx43 during the cell cycle [85,86]. Similarly, to serine phosphorylation, tyrosine phosphorylation has been correlated with the inhibition of GJ channels. For example, phosphorylation of Tyr247 and Tyr265 by the tyrosine kinase 2 (TyK2) leads to an overall increase of intracellular levels of Cx43 with decrease levels of Cx43 in the cell membrane [87]. In addition, it has been observed that TyK2 also indirectly participates in the phosphorylation of Ser279 and Ser282 by MAPK and Ser368 by PKC [87]. Phosphorylation of Cx43 at different sites controls the interactions with other proteins, GJ assembly, and turnover. There is then a large number of dynamic processes that are regulated by kinase-mediated signaling pathways. Figure 4 shows the main phosphorylation sites that can be present in Cx43 CT. Phosphorylation of Cx36, which is the major GJ component of electrical and mixed synapses in the CNS, can occur via calcium/calmodulin-dependent kinase II (CaMKII), which plays an important role in the activity-dependent plasticity of electrical synapses [88,89]. Two-putative CaMKII-binding sequences of Cx36, located at the cytoplasmic loop and CT of Cx36, have been reported [88]. It has been suggested that Cx36 follows a similar way of phosphorylation of glutamate receptors by CaMKII, where CaMKII binds to the NR2B subunit of NMDA receptors [88]. Further studies on Cx36 phosphorylation have been conducted on its homologous in fish, Cx35, where its phosphorylation state changes with conditions that change coupling. For instance, in retinal neurons, phosphorylation-dependent changes in coupling are driven by light adaptation or circadian rhythms [90,91]. Phosphorylation of the homologous Cx36 causes uncoupling and was found to be carried out in Ser-110 in the intracellular loop and Ser-276 in the CT by the protein kinase A (PKA) and the cGMP-dependent kinase (PKG) [92,93]. 

Another PTM is S-nitrosylation, in which nitric oxide (NO) binds to a reactive cysteine thiol to produce an S-nitrosothiol [94]. S-nitrosylation of Cx43 CT, which constitutively occurs on Cys-271 at GJ formed between endothelial cells and vascular smooth muscle cells [95], has been associated with a high cell permeability due to opening of Cx43 HCs. The suggested regulatory mechanism of Cx43 HCs permeability involves their dephosphorylation or modifications in cell oxidative environments due to the presence of reactive oxygen species (ROS), including NO [96]. Indeed, in cortical astrocytes with induced metabolic inhibition, it was observed an increment in cell permeability, which correlated with Cx43 S-nitrosylation along with Cx43 dephosphorylation [97]. On the other hand, Cx S-nitrosylation has been proposed to indirectly regulate Cx acetylation based on the relevance of NO to regulate histone deacetylases (HDACs) [98]. In contrast, the presence of oxidative stress has been associated with increased acetylase activity [99]. Putative lysine N^ε^-acetylation of Cx43 occurs on Lys-9 in the N-terminus and Lys234 and Lys264 in the CT [100]. In cardiomyocytes, it has been shown that Cx43 acetylation controls its subcellular localization [100]. A recent study showed in vivo that proper acetylation of Cx43 in the developing cerebral cortex requires of the Elongator complex [101], which is critical for controlling cortical neuron migration [102]. Cx43 thus interacts with the Elongator subunits, Elp1 and Elp3 [101], where the last subunit contains an acetyltransferase domain in its enzymatic core [103]. In addition, this acetylation in Cx43 is removed by HDAC6 [101].

Ubiquitination and SUMOylation are PTMs that can also be present in Cx. Transfer of the ubiquitin protein to a specific lysine residue on target proteins for degradation by an ATP-dependent process occurs via the ubiquitin–proteasome pathway that comprises E1 ubiquitin-activating enzymes, E2 ubiquitin-conjugating enzymes, and E3 ubiquitin ligases [104]. Similarly, lysine residues can be post-translationally modified by the small ubiquitin-like modifier (SUMO) family of proteins that are involved in multiple cellular processes like transcription, translation, cellular transport, cell growth, and programmed death [105]. In rodent brains, E3 ubiquitin ligases LNX1 and LNX2 localize at neuronal GJ formed by Cx36 [106]. Similarly, in the neuroblastoma cell line N2A transfected with a fluorescently labeled Cx36, both LNX1 and LNX2 interact with the Cx36 CT [106]. A significant reduction in neuronal Cx36 GJ was observed as a response to Cx36 ubiquitination. It was also suggested that this PTM has an important role in controlling the plasticity of electrical synapses formed by Cx36-containing neuronal GJs [106]. Similar to Cx36, Cx43 can be ubiquitinated, although the ubiquitination sites on Cx43 remain elusive [77]. Cx43 CT has ten lysine residues that can be potentially ubiquitinated. Different numbers of ubiquitin moieties can be conjugated to Cx43. This depends on several factors, such as cellular localization of Cx43 and cell type [81]. For instance, it has been found in epithelial liver cells, specifically in the rat cell line IAR20, that Cx43 can be modified with one up to four ubiquitinations at basal conditions [77].

Meanwhile, it has been detected two ubiquitination sites in exogenously expressed Cx43 from C6 rat glioma cells, which are similar to the ubiquitination pattern observed in mouse astrocytes [107]. It has been proposed that ubiquitinated Cx43 can recruit different ubiquitin-binding proteins, leading to its degradation via either autophagosomal pathway by interacting with proteins Eps 15 and p62, or endolysosomal pathway by interacting with Tsg101 [77]. Furthermore, inflammatory conditions activate the c-jun N-terminal kinase (JNK)-dependent ubiquitin-proteasome system in spinal astrocytes, leading to reduce levels of Cx43, where astrocytic GJ expression and function is disrupted [108]. On the other hand, Cx43 can be post-translationally modified by the small ubiquitin-like modifier (SUMO) family of proteins. In Cx43, membrane-proximal lysines, located in the intracellular loop and CT at positions 144 and 237, act as SUMO conjugation sites [100]. Opposed to Cx43 ubiquitinated, Cx43 SUMOylated helps to stabilize Cx43 at the plasma membrane [109]. Then, a crosstalk between Cx43 ubiquitination and SUMOylation to control Cx43 GJ endocytosis and degradation has been suggested. 

## 4. Connexins in Diseases of the Nervous System 

### 4.1. Role of Connexins in Glioblastoma

Glial cells fulfill a fundamental role in neuronal homeostasis maintenance and synaptic process development. This is especially true for astrocytes, where expression of Cx30, Cx36, Cx43, and Cx46 regulates synaptic neurotransmission, neuronal plasticity, liberation and uptake of neurotransmitters, inter-cellular communication, and cellular differentiation and growth [24,110,111]. However, changes in Cx expression and activity, which are thought to be modulated by pro-inflammatory cytokines, have been detected during illness-related processes [112,113]. This could have significant implications during cancer progression and treatment as both processes increase pro-inflammatory factors [114,115], which may explain cancer-related changes on Cx expression levels, cell localization, and PTMs like phosphorylation [116]. 

Gliomas are the most common type of brain cancer and have been histologically divided into four types: diffuse astrocytoma, oligoastrocytoma, oligodendroglioma, and glioblastoma [117]. Glioblastomas (GBMs) are considered the most aggressive, hardest to treat, and with the poorest prognosis. GBMs are characterized by a high degree of cellular heterogeneity and self-renewing tumorigenic stem cells that contribute to tumor propagation [118,119,120], therapeutic resistance [121,122], and low levels of Cxs [71,123,124,125,126]. Acquisition of a GBM malign phenotype may be favored by the reduction in Cx expression, which in turn has been hypothesized to have a regulating role in GBM development [116]. It is then possible that the restoration of normal levels of Cxs has antitumor effects [111,127]. However, it has been reported that hetero-cellular GJIC between glioma cells and their surrounding environment enhances their progression by protecting tumor cells through the distribution of therapy-related degradation [128,129], suggesting that GJ downregulation or Cx targeting could contribute to GBM treatment [130,131].

Several factors have an impact on the activity of Cxs during neoplastic disease in glial cells such as Cx subtype, aging, glioma subtype, cancer stem cells, differentiation, phosphorylation state, localization, and tumor malignancy. Tumors can be affected by Cxs in different ways according to their isotype and tumor type [127]. For example, Cx30 is present in mature astrocytes, older than 12 weeks, but absent in rat gliosarcoma 9L and rat glioma C6 cells. However, restoring Cx30 levels in these glioma cells has anti-tumoral properties [111,132]. In contrast, it has been found that Cx30 can protect gliomas from radiation therapy, causing an inverse correlation between the patient’s prognosis and the expression level of this Cx [132]. Although little is known about Cx36, recent studies have reported its expression in rat brain microglia [133] and human astrocytic brain tumors (Grade II, Grade III, and Grade IV) [134]. In astrocytic tumors, Cx36 increases its levels inside tumor cells but decreases its expression in the environment depending on the tumor grade [134], which makes Cx36 a useful biomarker for tumor diagnosis and prediction of neoplasm progression. However, whether Cx36 increases as a compensatory or pathological mechanism is unclear [135]. Hereof, more studies are needed to elucidate the conditions and mechanisms that regulate Cx36 expression in astrocytic tumors because not all glioma cells express Cx36, like the glioma cells F98 [131]

Although Cx30, Cx36, and Cx43 are the Cxs with more information available for GBM, they also have a high number of contradictory findings reported [136]. On one side, some studies have related the absence of Cx43 with tumor grade increase, associating this Cx with neoplastic tumor processes. However, other studies suggest that the absence of Cx43 does not necessarily lead to tumor formation [137,138]. Similarly, some studies reported that the increase in Cx43 levels could inhibit tumor capacity of self-renewal, invasiveness, and/or tumorigenicity [126]. Nonetheless, other studies have suggested that Cx43 can increase the migration of some tumors based on the cell type, despite blocking their proliferation [138]. It is hypothesized that the reason behind all these contradictions is the inadequate number of samples on Cx43 studies, which reduce their accuracy, given the high cell heterogeneity present in GBM [139].

As mentioned before, GBM is a highly heterogeneous disease, so new classifications have been created due to the different behaviors observed for this pathology [117,140,141]. It has been considered that the amount of Cx43 decreases according to the progression of the tumor [116,139]. However, it is necessary to specify the cell line or GBM classification. For example, Cx43 is expressed in 9L cells but is less abundant in C6 cells [111]. Likewise, it is essential to consider the sample size; for example, in a study with 85 samples (37 grade IV, 18 grade III, 24 grade II, and 6 grades II to III), it was found that Cx43 was expressed in more than 60% of glioblastomas [139], evidencing the large heterogeneity of the disease. Another factor that affects Cx expression is aging. For instance, changes in the type and mRNA levels of Cxs have been observed between mouse embryos and neonates [142]. During brain development, in mouse E9.5 and E10.5 embryos, Cx26, Cx29, Cx30, Cx30.3, Cx32, Cx36, Cx46, and Cx47 are expressed. However, in addition to the previous Cxs, mRNA of Cx37, Cx43, Cx45, and Cx59 are also found in mouse neonatal brains but Cx30.3 mRNA is not further detected [142]. Moreover, the expression level and distribution of these Cxs in neonatal brains vary. For example, Cx29 is highly expressed in myelinating glia and acoustic nerve, Cx43 is found in meninges and astrocytes, while Cx47 is found in distinct cells in the brain and cranial nerves [142]. It has also been found that Cx30 is not expressed in astrocyte culture until several weeks [137]. Studies of Cxs in cell culture models require then a strict control of animal age and culture time. 

Furthermore, different correlations between Cx expression and glioma subtype have been reported. For instance, a negative correlation was found between GBM patient survival and Cx46 expression [127], while in the proneural molecular subtype, this negative correlation is related to Cx43 expression [125]. In the proneural subtype, this effect is caused because the platelet-derived growth factor (PDGF) signaling inhibits GJs, generating selective pressure on the type of Cx expressed [125]. Cancer stem cells (CSCs) represent a severe problem in public health due to their drug resistance, invasiveness, tumorigenicity, ability to self-renewal, induction of cell cycle arrest, and differentiation into heterogeneous lineages of cancer cells [121,143,144]. Recent studies have found that there is a difference in the expression of Cxs present in CSCs and non-CSCs, so it is crucial to keep in mind that the disease per se is not the only variable to be considered when studying Cxs in glial cells [127]. For example, although CSCs express low levels of Cx43 [124,126], they also express high levels of Cx46 [127], which is a CSC protector. Another factor affecting Cx expression is cell differentiation. For example, during this process, there is an increase in Cx43 and a decrease in Cx46 expression [127]. Though the regulation between Cx46 and Cx43 has been reported [145], according to recent studies, this is not direct [127]. It has been suggested then, for the treatment of CSCs, not only to increase Cx43 via transfection or with mimetic peptides [146] but also to perform a direct block of Cx46 to increase the treatment efficiency in CSCs.

Phosphorylation of Cxs, mainly reported for Cx43, is a fundamental regulation process during disease progression. Many growth factors, oncogenes, and tumor-promoting chemicals can phosphorylate Cxs, which has been associated with autophagy degradation [77,147], inhibiting GJICs [148]. Aberrant cellular localization of Cxs plays an important role in glioma cell growth. For example, the nuclear localization of Cx30 has been associated with reduced glioma cell growth due to limited GJIC [149]. Suggesting a possible role of Cxs in the regulation of gene expression [149]. On the other hand, Cx translocation to mitochondria has been observed as a response to cellular stress, like for Cx43 [132]. However, translocation of Cx30 to mitochondria favors ATP production, DNA repair, and cell survival, protecting glioma cells from radiotherapy [132]. Furthermore, functional changes in Cx43 have been observed depending on the cell type, malignant or non-malignant tumors, as well as with tumor grade. For instance, in high-grade glioma, there is a decrease in intercellular communication [150,151]. This may be because although RNA is still produced for the synthesis of Cx43, it does not necessarily end up with the formation of functional GJICs.

Although there is extensive evidence that correlates alterations in the epigenome with cancer establishment, progression, and acquisition of characteristic hallmarks, epigenetic changes associated with Cx expression in brain cancer is mainly limited to the post-transcription regulation exerted by miRNAs (see Section 2.2). This undoubtedly shows the need for studies that provide experimental evidence of the epigenetic mechanism involved in the regulation of Cxs expression associated with brain cancer.

### 4.2. Connexins in Neurodegeneration

**Figure 5:**
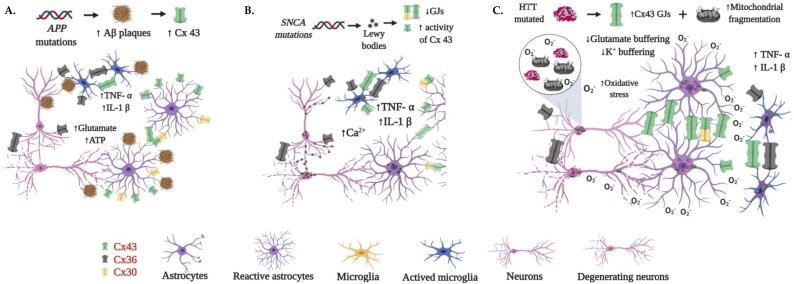
Schematic representation of main perturbations in the expression of Cx30, Cx36, and Cx43 in neurons, astrocytes, and microglia for (**A)** AD, (**B**) PD, and (**C**) HD. Common features are observed across the different neurodegenerative disorders such as reactive astrocytes, active microglia, the release of pro-inflammatory molecules (*e*.*g*., TNF-α and IL-1 β), loss of glutamate and K^+^ buffering capacity, and generation of ROS. Briefly, in AD (**A**) mutations in the *APP* gene leads to the accumulation of Aβ plaques, which is associated with increased levels of Cx43 and chronical activation of Cx43 HCs. In PD (**B**), α-synuclein enhances the opening of Cx43 HCs, leading to high intracellular Ca^2+^ levels along with the activation of cytokines. In HD (**C**), abnormally long polyglutamine in HTT protein causes mitochondrial fragmentation, which has been mainly associated with increased Cx43 GJs.

Neurodegenerative diseases, such as Alzheimer’s disease (AD), Parkinson’s disease (PD), and Huntington’s disease (HD), are characterized by reactive gliosis, which is associated with phenotypic changes in astrocytes and microglia [152]. In this sense, astrocytes respond during neurodegenerative processes by releasing different molecules such as neurotrophic, inflammatory factors, and cytotoxins [152]. These responses have been associated with modifications in the expression and function of Cxs [153]. Despite the acknowledgment of the role played by Cxs during the onset and progression of neurodegenerative disorders, there is a gap in the knowledge of how the expression of Cxs is perturbed. We provide here the most relevant findings associated with the aberrant expression of Cx30, Cx36, and Cx43 in AD, PD, and HD, which could encourage to study the regulation in the expression of Cxs during the onset and progression of these disorders. The main findings related to neurodegeneration associated with aberrant expression or function of Cx30, Cx36, and Cx43 in AD, PD, and HD are summarized in Figure 5.

#### 4.2.1. Alzheimer’s Disease

Alzheimer’s disease (AD) is characterized by progressive and chronic learning and memory loss and changes in mood and behavior [154]. Genetically, AD is divided into familial and sporadic forms. The familial form is characterized by mutations in the amyloid precursor protein (*APP*) gene, presenilin1 (*PSEN1*) gene, and presenilin 2 (*PSEN2*) gene. In the sporadic form, which results from a combination of genetic and environmental factors, the inheritance of the apolipoprotein E ε4 (*APOE4*) allele is considered a risk factor for developing AD [155,156]. Hallmarks of AD are neuritic plaques, neurofibrillary tangles, and neuronal loss. However, the prominent pathological feature of AD is the formation of amyloid plaques, which are deposits of different sizes of small peptides called β-amyloid (Aβ). These Aβ plaques are derived via sequential proteolytic cleavages of the Aβ precursor protein (APP) by the action of β- and γ-secretases [154,157]. Among the different Aβ fragments, Aβ_1-40_ and Aβ_1-42_ are thought to be critical elements in AD pathogenesis due to their neuropil and vascular accumulation [158]. However, several synthetic Aβ fragments have been used in in vitro neurotoxicity studies. Among these fragments, peptide Aβ_25-35_ has been widely used, which is the shortest fragment of Aβ found in vivo and is considered the biologically active region of Aβ_1-42_ [159]. Although Aβ plaques are considered crucial in AD pathogenesis and pathophysiology, they do not reflect the clinical progression of AD [158]. It has been hypothesized that the soluble Aβ oligomers (AβOs) are the most toxic and pathogenic form of Aβ, characterized for being >50 kDa, reactive to the anti-amyloid oligomer antibody A11, and unrelated to amyloid plaques [160]. Among the different toxic effects detected in vitro and in vivo, AβOs bind to the cellular prior protein receptor [161], induce the production of ROS [162] and disrupt memory function [160]. Notwithstanding the evidence of AβOs role in AD pathophysiology, there is no consensus regarding the molecular form(s) of Aβ responsible for the neurological decline in AD patients. The reader is referred then to recent and comprehensive reviews on AβOs [158,160,163].

Recent studies have achieved breakthroughs in establishing communication between different glial cell types and the onset and progression of AD via Cx expression and modulation [164,165]. In AD patients and murine animal models of AD, it has been demonstrated that GJ and Cx are mainly overexpressed in astrocytes [164,166]. In post-mortem temporal cortex samples of AD patients, it was observed a large amount of Cx43 in astrocytes that were positive for glial fibrillary acidic protein (GFAP). These astrocytes also presented enlarge cell bodies and relatively short and thick processes that are characteristic of reactive astrocytes. In addition, about 80% of these Cx43 patches co-localized with Aβ plaques, suggesting a relationship between GJ intercellular interaction and diverse roles of APP [166]. In agreement with these findings in AD patients, it was found in an AD mouse model that Cx43 HCs were chronically activated in hippocampal astrocytes when Aβ plaques were present [165]. Furthermore, astrocytes that were in contact with plaques showed a prominent activation of HCs and high Cx43 content [165]. Similarly, in an APP x PS1 transgenic mouse model, an increased immunoreactivity of Cx43 and Cx30 in hippocampus cells that largely expressed the GFAP was observed, which is characteristic of dysfunctional reactive astrocytes that are in contact with amyloid deposits [167]. Moreover, KO of the Cx43 gene in APP x PS1 transgenic mouse reduced neuronal damage by decreasing neuritic dystrophy and oxidative stress [165]. In addition, it was reported that Cx43 promotes the survival of adult-born neurons in mouse hippocampus, while their survival is restricted under Cx30 expression [168]. These shreds of evidence support the critical and complex role that Cx43 HCs have in neuronal damage in cortex and hippocampus during AD progression. 

Contrary to Cx43 and Cx30, which have been studied in murine AD models, the study of Cx36 in AD has been limited to in vitro assays [169]. Cultured neurons exposed to the acute application of Aβ_25-35_ showed increased activity of Cx36 HCs. However, compared to the control, no significant difference was observed in the levels of surface and total Cx36. It was then proposed that the increment in HCs activity associated with Cx36 can be owed to an enhanced channel permeability result of PTMs of the Cx [40]. Figure 5A summarizes the most relevant findings associated with the dysregulation of Cxs in AD.

#### 4.2.2. Parkinson’s Disease

Parkinson’s disease (PD) is the second most common neurodegenerative disease after AD [170,171]. PD is a complex, progressive, and neurodegenerative disease characterized by loss of dopaminergic neurons (DNs) in the substantia nigra pars compacta (SNc), along with the formation of cytoplasmic inclusions of α-synuclein (called Lewy bodies) [172]. PD is characterized by a widespread pathology that can involve other brain regions and non-dopaminergic neurons [173]. Its principal motor symptoms caused by loss of DNs along with dopamine depletion are tremor, rigidity, bradykinesia/akinesia, and postural instability, but the clinical picture includes other motor and non-motor symptoms (NMSs) [174]. It is known that most cases of PD have a multifactorial etiology as a result of the combination of environmental and genetic factors. For example, exposure to toxic chemicals such as pesticides, herbicides, and heavy metals [175,176,177,178] or head injuries can increase the risk of suffering PD [179]. In PD, it has been observed the accumulation of α-synuclein in astrocytes, which results in microglial activation due to the release of pro-inflammatory cytokines, such as tumor necrosis factor-α (TNF-α), interleukin-1β (IL-1β) and interferon-gamma (IFN-γ) [180]. In this sense, microglial activation has been proposed to be beneficial in the early stage of the neurodegeneration process, where microglial cells attempt to clear α-synuclein from the extracellular space as a result of cellular releases or apoptotic neuron death [181]. However, long-term activation of microglia significantly increases the levels of pro-inflammatory cytokines and ROS leading to a deterioration of the neurodegenerative process [181].

On the other hand, almost 20 genes related to PD have been identified. However, most of the animal models carrying mutations in these genes have failed in the development of PD phenotypes, those carrying mutations in the α-synuclein (*SNCA*) gene being the exception [182]. The dysfunction of astrocyte and microglia makes the brain microenvironment poor for neuron survival, and it has been proposed to be a more plausible mechanism for the gradual neurodegeneration observed in PD patients [182]. Astrocyte dysfunction has been associated with the accumulation of α-synuclein, which leads to a severe loss of DNs [183], increased expression of S100β that favors the activation of receptors of inflammatory mediators such as TNF-α [183], and a reduced presence of positive astrocytes for glutathione peroxidase that is related with the hyperoxidation phenomena [184]. Furthermore, α-synuclein has been reported to enhance the opening of Cx43 HCs in cortical astrocytes, leading to the increment of intracellular Ca^2+^ along with the activation of cytokines, cyclooxygenase 2, and inducible nitric oxide synthase [185]. Figure 5B depicts some of the most relevant findings related to the dysregulation of Cxs by α-synuclein.

To establish whether Cx-mediated HCs play a crucial role in this astrocyte dysfunction, animal models in which PD have been chemically-induced are used. 6-Hydroxydopamine (6-OHDA), 1-methyl-4-phenyl-1,2,3,6-tetrahydropyridine (MPTP), and rotenone are the most common neurotoxic molecules used to induced degeneration of DNs [186]. The use of MPTP causes a pronounced immediate, but transient increase in striatal Cx43 mRNA, which was paralleled to a sustained increase in Cx43 immunoreactive puncta [187]. However, the increase in Cx43 immunopositive puncta was not paralleled by alterations in the functional coupling of striatal glial cells [187]. Similarly, in an in vitro and in vivo rotenone-induced model of PD, increased levels of Cx43 were observed along with and increased GJIC, which was further verified in a rat PD model with the increment of phosphorylated Cx43 that is required for GJIC. [188]. This enhancement in total and phosphorylated Cx43 was observed in the striatal and hippocampal regions. These results suggest an enhancement of GJIC through the induction of phosphorylated Cx43 as well as total Cx43 in astrocytes [188]. This upregulation in Cx43 expression led to elevated HC activity, enhanced GJ coupling, and increased intracellular Ca^2+^ concentration, which contributed to neurotoxicity. Nevertheless, another study using rotenone showed down-regulation in Cx43 expression and decreased GJ permeability in primary cultured astrocytes, which reveals that the dysfunction of astrocytic GJ may be implicated in the PD pathology [189]. In contrast, pretreatment with the selective mitochondrial ATP-sensitive potassium (K_ATP_) channel openers Iptakalim (10 μM) or Diazoxide (100 μM) prevented rotenone-induced down-regulation of Cx43 and the loss of GJ permeability [189]. These effects were abolished when 5-HT hydroxydecanoate, a mitochondrial K_ATP_ channel blocker, was used [189]. These results suggest that opening mitochondrial K_ATP_ channels in astrocytes may protect against rotenone-induced dysfunction of astrocytic Cx43. 

On the other hand, a study in a 6-OHDA-induced rat model of PD showed that levels of Cx30, but not of Cx43, were increased in the striatum; meanwhile, around vessels, the levels of Cx43 and Cx30 were largely increased, suggesting an increased metabolic coupling [37]. Then, it is suggested that in induced parkinsonism, astrocytosis is massive and is not associated with an increased coupling between astrocytes, but might be associated with an increased metabolic coupling [37]. However, in an acute PD mouse model generated by MPTP administration, Cx30 and Cx43 were upregulated in the striatum [38]. In this study, it was assessed if Cx30 overexpression could influence the expression of Cx43, finding no correlation between Cx30 overexpression with either the levels or distribution of Cx43 upon MPTP treatment. In addition, in a Cx30 KO model, it was shown that MPTP treatment accelerates the loss of DNs, reduces the up-regulation of the S100a10 gene, which is important for cell migration and intracellular trafficking, and partially suppresses the GFAP and astrogliosis in the striatum [38]. These results suggest that the neuroprotective function of astrocytes in the striatum is diminished upon a deficiency of Cx30, for which enhancement of Cx30 functions is proposed as a therapeutic strategy for PD patients. Contrary to previous observation for Cx30 in a 6-OHDA-induced rat model of PD, it was observed reduced levels of Cx36 in the cerebral cortex and striatum when compared to the control group [190]. However, the 6-OHDA effect was partially reversed by Baicalin, suggesting a neuroprotective effect of this molecule. 

Cx36 GJ channels have different regulatory properties compared to other Cx isoforms, such as low unitary conductance and sensitivity to transjunctional voltage; perturbations associated with these regulatory functions have also been explored in PD [191,192]. Changes in the discharge of the basal ganglia, such as increased synchrony, burst discharges, and β-oscillations, have been recorded in PD patients [193]. It was then hypothesized that GJs could be involved in the intrinsic mechanism of synchronization due to a possible GJ remodeling and GJ coupling associated with dopamine levels, in which GJ conductance is reduced when dopamine levels are increased [194]. Using putamen and external and internal globus pallidus tissue from PD patients, it was found that Cx36 GJs were numerous and high in conductance when compared to their counterparts in control subjects. In contrast, no Cx36 was found in the human PD subthalamic nucleus. Therefore, it has been proposed that Cx36 GJs act as a synchrony modulator in the basal ganglia [195]. On the other hand, attenuate β-oscillations and improve forelimb function has been observed when unspecific GJ blockers were used in hemiparkinsonian rats [196], suggesting a global contribution of GJ to β-oscillations. 

Overall these observations show the different effects that induced-PD via chemical treatments have over the expression and function of Cx30, Cx36, and Cx43. Then, this may be relevant during the evaluation of candidate molecules to ameliorate cognitive and motor impairment in PD. 

#### 4.2.3. Huntington’s Disease 

Huntington’s disease (HD) is a hereditary neurodegenerative disorder characterized by progressive motor, behavioral, psychiatric, and cognitive decline, ending in death [197]. The inheritance pattern of HD is autosomal dominant with the onset of symptoms usually occurring in the third or fourth decade of life, in which patients present chorea, or involuntary movements, and/or behavioral changes [198]. HD is caused by a dominantly inherited trinucleotide cytosine-adenine-guanine (CAG) repeat expansion in the huntingtin (*HTT*) gene, resulting in a mutant HTT protein with an abnormally long polyglutamine repeat [199]. Changes in mitochondrial morphology, fusion/fission imbalance, and oxidative DNA damage have been related to the degeneration of striatal and cortical neurons [200,201]. Furthermore, mutant HTT is associated with decreased respiratory function as well as with changes in mitochondrial mobility and ultrastructure [202,203]. 

Like in AD and PD, abnormal astrocyte function contributes to HD pathology. In cortical and striatal astrocytes from HD patients and animal models, it has been found an accumulation of mutant HTT protein, which disrupts astrocyte glutamate transporter expression [204,205]. During early HD stages, several astrocyte dysfunctions have been revealed: (i) reduction of K^+^ buffering mediated by decrease Kir4.1 channel expression in two mice models (R6/2 and Q175) for HD in striatal astrocytes; and (ii) disrupted intracellular Ca^2+^ signaling and decreased extracellular uptake of glutamate GLT1-mediated [206,207,208]. The increase in extracellular K^+^ levels, in turn, results in the intensification of depolarization that may further underlie the hyperexcitability of striatal medium spine neurons (MSNs) in the striatum [208]. In addition, astrocytes exhibited profound morphological deficits, including reduced volumes of peri-synaptic astrocytes processes, territory size, and altered proximity of astrocyte processes to cortical and thalamic excitatory entrances, revealed by the neuron-astrocyte proximity assay (NAPA) [205].

Different animal models have been developed for HD following genetic and non-genetic strategies [209]. HD-induced models based on genetic strategies, such as transgenic and KO rodent models, mimic in a better way the HD progression and pathology than non-genetic strategies that are based on 3-nitropropionic acid or quinolinic acid treatments [209]. In this sense, the transgenic R6/2 mouse, which expresses exon 1 of the human *HD* gene with up to 157 CAG repeats as HD model, has been used to study the role of Cxs in the visual system [210]. Given the abnormalities observed in the visual system that affects the visuomotor cognition in HD patients [211], the role of Cx36, the main Cx in retina, has been studied. In retinal degenerations, it was found that Cx36 was slightly decreased in the external plexiform layer in the HD model compared to control subjects. Therefore, it has been suggested that Cx36 is related to the degeneration of photoreceptor terminals [212]. 

Excitatory and inhibitory inputs in MSNs are affected by the interneuronal connectivity mediated by Cx36 GJs since Cx36 KO in a rodent model induced a reduction in both excitatory and inhibitory postsynaptic currents [213]. In this sense, the frequency of excitatory and inhibitory postsynaptic currents in two transgenic murine models for HD, one containing the full human *HD* gene with 128 CAG repeats and the other one containing a chimeric mouse/human exon 1 containing 140 CAG repeats inserted into the murine *Hd* gene [214], was studied. Then, MSNs from both HD animal models showed a reduction in the frequency of excitatory and inhibitory postsynaptic currents compared with those observed in MSNs from wild type animals [214]. Putting together these reports, it is likely to then expect a reduction in Cx36 GJs that affect the synchrony in MSNs from HD animal models.

On the other hand, the distribution of different Cxs, such as Cx26, Cx43, and Cx50, in the caudate nucleus (CN) and globus pallidus (GP) of the basal ganglia in healthy and HD human brains was reported [215]. In this study, no significant difference was observed in the levels and distribution of Cx43 GJs in GP from both healthy and HD brains. However, HD brains had increased levels of Cx43 GJs in the CN than normal brains, and these were localized in patches. This correlates with the increased level GFAP immunoreactivity in astrocytes in CN compared to healthy brains. This pattern manifested reactive astrocytosis around degenerating neurons with increased expression of astrocytic GJs. If small units are supposed to be functional, it could suggest an improved coupling status between astrocytes, which could provide a higher capacity for spatial damping by astrocytes in an attempt to maintain an adequate environment for neurons, helping to promote neural survival in HD [215]. 

Despite these observations that showed the aberrant expression of Cx36 and Cx43 in HD animal models and human brains, there is little information that allows the understanding of the pathophysiology of HD and the role of Cxs and astrocytes in maintaining homeostasis in the HD. In addition, no one, to the best of our knowledge, has studied the Cx30 changes in HD. Figure 5C shows the most relevant findings associated with the dysregulation of Cxs in HD.

## 5. Connexins in Neuroprotection

Cxs are essential to maintain homeostasis of the CNS, and changes in their function and expression have been linked to neuroprotective mechanisms in ischemia [216,217,218], traumatic brain injury (TBI) [219], and glaucoma [220]. In these kinds of injuries, intrinsic mechanisms regulate primary cell death, while cellular intercellular communication seems to play a significant role in secondary cell death by regulating the pass of toxic molecules [221]. However, there is still controversy in the field because it is not clear if indeed Cxs are neuroprotective or stimulate neurodegeneration, and whether glia and neuron communication or a combination of both contributes to neuroprotection.

In ischemic strokes, neurons and astrocytes are rapidly depleted of energy and oxygen, a process that ultimately leads to neuronal loss viability, irreversible tissue damage, and neurological deficits. Cx43 is highly expressed in astrocytes, and it has been implicated in the formation of a functional syncytium that buffers ions, proteins, and other molecules from the extracellular space [218]. Different studies suggest that Cx43 responds to physical injuries or cytotoxic glutamate levels by enhancing GJIC and glutamate uptake [218,222]. In agreement with the critical role of this Cx in homeostasis, Cx43 KO mice show astrocytic impaired Ca^2+^ signaling and GJ uncoupling that induces neuronal cell death and accumulation of K^+^ ions and glutamate in the extracellular space [223,224]. Consequently, deficiencies in the glial syncytium function in the Cx43 KO mice may perhaps explain the observed increase in the brain infarct volume after a middle cerebral artery occlusion (MCAO), suggesting a neuroprotective role of GJIC and this Cx in ischemia [218]. Cx43 is not only expressed in astrocytes, but it is also found in ependymal, leptomeningeal, and vasculature cells [216,217], suggesting that in Cx43 KO mice, loss of function of this protein in other cells than astrocytes could help to extend the infarct zone and increase apoptosis during ischemia. However, studies using conditional KO mice that only affect the expression of Cx43 in astrocytes found that a focal stroke also extends neuronal apoptosis and infarct zone inflammation, linking the expression of this Cx in astrocytes to the resolution of ischemia and neuroprotection.

Mechanistically, a decline in astrocytic GJIC can lead to a reduction in the propagation of a stroke injury by regulating apoptosis and gliosis [216]. For instance, in ischemia, apoptosis is increased by a blockage in blood supply that generates a massive release of glutamate and Ca^2+^ influx that is followed by the production of ROS that further increases cytotoxicity. As a result, in the Cx43KO mouse model, apoptotic signals such as caspase-3 and cytochrome C are not efficiently removed and remained high for more extended periods, affecting cell viability. Remarkably, the Cx43 surface expression is differentially altered at the core and penumbra. At the core, Cx43 is internalized, while at the penumbra, its expression remains stable [217,225]. This suggests that at the core, the downregulation of Cx43 impairs the diffusion of apoptotic signals such as Ca^2+^, and ROS among others [226,227], whereas at the penumbra facilitates the removal of cytotoxic molecules that may affect the ischemic lesion volume [217].

The neuroprotective role of astrocytic GJIC and Cx43 has been controversial and challenged by in vitro ischemia studies using gap-junctional blockers such as carbenoxolone (CBX), octanol, and halothane that showed not only a decreased infarct volume but also a significant reduction in neuronal cell death [226,228]. Although the causes of these differences have not been fully established, these conflicting results can be explained by several factors such as

*i*. *Blockers unspecificity.* Besides blocking GJIC, CBX, octanol, and halothane have been shown to interfere with excitatory synaptic transmission and voltage-gated Ca^2+^ channels activity [226], phenomena that per se can further decrease cytotoxicity and cell death. 

*ii*. *Reduced spreading of stress signals*. During ischemia, the accumulation of pathogenic factors can be easily spread through GJs, which in turn can amplify the pathologic processes to areas beyond the injury site, a process that can be blocked more efficiently by GJIC inhibitors that can affect a broad type of Cxs [229].

*iii*. *Differential and broad Cx expression in the CNS.* Neurons different from astrocytes express a plethora of Cxs such as Cx26, Cx32, and Cx36 that can be differently regulated during ischemia. The use of specific antisense probes against Cx32, Cx26, and Cx43 on in vitro ischemia paradigms have shown that the silencing of Cx26 and Cx32 simultaneously, or of Cx43, significantly reduces cell death [229]. This has been further corroborated in models of traumatic brain injury using organotypic hippocampal slices, Cxs inhibitors, antisense Cxs oligodeoxynucleotides, or Cx43 KO decreased cell death and alleviated synaptic function impairments [230]. Likewise, excitotoxic and ischemia retina models have shown that knocking-out Cx36 increased cell survival under excitotoxic stimuli but did not protect ischemic cells [221]. Similarly, hippocampal GABAergic neurons co-expressing alpha α7-subunit nicotinic acetylcholine receptor and Cx36 are more resistant to oxygen-glucose deprivation that pyramidal neurons [231]. Hence, Cx-mediated neuroprotection can be regulated by the differential expression of Cxs in neurons and astrocytes, communication between glia and neurons, and type of injury.

*iv*. *Role of GJIC and Cx HCs*. During ischemia, Cx HCs can open, and blockade of Cx43 HCs is protective. The use of mimetic peptides that blocks Cx43 HCs revealed that Cx HCs opening after ischemia contributes to the injury spread by the release of neurotoxic molecules, glutamate, and ATP [228,232]. ATP released from astrocytes is an important regulator of microglial activation by inducing the release of pro-inflammatory cytokines from these cells. These cytokines then act back on astrocytes, reducing the levels of GJIC and increasing astrocytic HCs [232]. Therefore, it is possible that in Cx43 KO, increased compensatory expression of other Cx HCs in astrocytes during ischemia can exacerbate the release of cytotoxic and pro-apoptotic molecules. In contrast, in ALS, a significant rise in Cx43 expression leads to enhanced GJIC coupling, increased HC activity, and elevated intracellular Ca^2+^ levels. Interestingly, the blocking of both GJIC and Cx43 HCs protected motor neurons, suggesting an essential role of Cx channels and HCs in the disease [233]. 

Despite the debate about the direct role of Cxs in neuroprotection, recent studies have found that Cxs may be a downstream target of endogenous and exogenous neuroprotective molecules. For instance, corticotropin-releasing hormone (CRH) is a regulator of MAPKs and protein kinase A-cAMP response element-binding protein, which in turn has been shown to regulate the activity of Cx43 [234,235]. In IMR32 cells, primary astrocytes, and organotypic hippocampal slice cultures, CRH treatment up-regulates the expression of Cx43 and the number and size of gap junctions [236]. Also, CBX inhibits the CRH-neuroprotective effect against the toxic amyloid peptide 25–35 fragment or H_2_O_2_ on neurons, suggesting that Cx43 expression in astrocytes is part of signaling pathways that regulate neuronal viability by regulating cytoskeletal rearrangements, gene expression, or reducing oxidative stress under stress conditions [236]. However, leptin, a neurotropic and anti-apoptotic hormone secreted by adipocytes, regulates neuroprotection by decreasing the expression of Cx43 HCs during MCAO or oxygen and glucose deprivation (OGD) by a mechanism that involves the activation of ERK1/2 MAP kinases [237].

Exogenous neuroprotective molecules such as ginsenosides (a secondary metabolite isolated from Panax ginseng) [238], tongxinluo (TXL, a multifunctional traditional Chinese medicine) [239], and baclofen (GABAb receptor agonist, muscle relaxer and antispasmodic agent) [224], regulate neuroprotection by controlling Cxs expression. In TBI, ginsenosides decreased the up-regulation expression of Cx40 induced by the trauma throughout the activation of the MAPK pathway [238]. Also, TXL significantly improved neurological deficit and reduced the infarction volume after an MCAO by increasing the expression of Cx43 and reducing the activity of apoptotic pathways (calpain II/Bax/ caspase 3 pathway) [239]. In a hippocampal atrophy model induced by chronic cerebral hypoperfusion, baclofen showed to decrease the expression of Cx43 and Cx36 in the membrane and mitochondria, enhancing autophagy mechanisms that induce neuroprotection [240]. Cxs are complex proteins, which have several phosphorylation sites in their CT that are important for junction assembly, activity, and interaction with other proteins [241]. For instance, studies using Cx43 CT truncated mice (Cx43ΔCT) showed that this region is sufficient to regulate infarct volume, astrogliosis, inflammatory invasion, and protection in cerebral ischemia [241]. Hence, it is likely that endogenous and exogenous molecules, which have neuroprotection activity through Cxs, work by regulating kinases that phosphorylate the CT of Cxs.

On the other hand, despite that Cx30 is one of the main Cxs in astrocytes, its neuroprotective properties in ischemic strokes is limited. To the best of our knowledge, only one study that evaluated the effect of hydrogen treatment on transient global cerebral ischemia (TGCI) assessed the changes in Cx30 [242]. In that study, hydrogen was used due to its anti-oxidative, anti-inflammatory, and anti-apoptotic effects in a TGCI rat model. Compared to the naïve control group, non-treated TGCI animals showed upregulated levels of Cx30 in the hippocampus, but in the cortex, no changes in Cx30 levels were observed. After hydrogen treatment, Cx30 levels were reduced in the hippocampus. This reduction in Cx30 GJs has been associated with a reduced injury induced by TGCI in rats [243], suggesting that in TGCI, Cx30 GJs blockers may act as neuroprotective molecules.

Although several steps have been given in understanding the role of Cxs in neuroprotection, there is still an ample space to fill regarding the signaling mechanisms that can lead to the differential action of neuroprotective molecules working throughout GJIC or Cx HCs.

## 6. Conclusions

Cxs, especially Cx30, Cx36, and Cx43, play an important role in keeping cell homeostasis in the CNS. Their deregulation has been related to different diseases, particularly to glioblastomas and neurodegenerative diseases such as AD, PD, and HD. In addition, they have also shown to be important in neuroprotection processes. Although there are still debates and contradictory findings of the up- or down-regulation effect of Cxs on neuroprotection during the onset and progression of GBMs as well as in neurodegenerative disorders, there are studies that have demonstrated that Cx GJs can be blocked to mitigate the neurodegenerative process. In this sense, the search for new molecules that can selectively target neuronal or glial Cxs is required to ameliorate their dysregulation during the onset and progression of these diseases. Reactive glia is one of the primary characteristics observed in both GBMs and neurodegenerative disorders. Focusing then on targeting Cx30 and Cx43 GJs in glial cells is a therapeutic approach in neurodegeneration. The current state of the art demonstrates that understanding the role Cxs and how changes in their expression emerge in neurons and astroglia during the onset and progress of neurodegenerative diseases is crucial for the establishment of alternative therapies. 

However, to fully understand their role in all these scenarios, it is necessary to elucidate their regulation at different stages. In this review, we present different molecular aspects related to the epigenetic regulation of Cx expression, such as DNA methylation, histone PTMs, and miRNA, as well as the main PTMs, which Cxs can have and affect their life cycle and protein interactome. Despite the acknowledgment of the role played by Cxs during the onset and progression of neurodegenerative disorders, there is a gap in the knowledge of how the epigenetic machinery behind Cx regulation is perturbed in these diseases. We provide then the most relevant findings associated with the aberrant expression of Cx30, Cx36, and Cx43 in AD, PD, and HD, which could encourage the study of the epigenetic changes related to Cx regulation in these disorders. Thus, further exploration of how epigenetic machinery affects Cx expression and channel activity and how Cxs are involved in signaling pathways will be, in the upcoming years, a promising opportunity for the therapy of brain cancer, neurodegenerative diseases, and neuroprotection.

## Abbreviations

AD: Alzheimer´s disease; APP, Amyloid precursor protein; Aβ, β-amyloid; CaMKII, Calcium/calmodulin dependent kinase II; CDK1, Cyclin-dependent kinase 1; CK1, Casein kinase 1; Cxs, Connexins; DNMTs, DNA methyltransferases; DNMTi, DNA methyltransferase inhibitor; GBMs, Glioblastomas; GFAP, glial fibrillary acidic protein; GJs, Gap junctions; GJIC, Gap junctional intercellular communication; HCs, Hemichannels; HD, Huntington´s disease; HDACs, Histone deacetylases; JNK, Jun N-terminal kinase; KATs, Lysine acetyltransferases; KMTs, Lysine methyltransferases; MAPK, Mitogen-activated protein kinase; PD, Parkinson´s disease; PDGF, Platelet-derived growth factor; PKA, Protein kinase A; PKC, Protein kinase C; PKG, cGMP-dependent kinase; Src, Src kinase; TBI, Traumatic brain injury.
